# One millennium of historical freshwater fish occurrence data for Portuguese rivers and streams

**DOI:** 10.1038/sdata.2018.163

**Published:** 2018-08-14

**Authors:** Gonçalo Duarte, Miguel Moreira, Paulo Branco, Luís da Costa, Maria Teresa Ferreira, Pedro Segurado

**Affiliations:** 1 University of Lisbon, School of Agriculture, Forest Research Centre, Tapada da Ajuda, 1349-017 Lisbon, Portugal; 2 University of Lisbon, Instituto Superior Técnico, CERIS-Civil Engineering Research and Innovation for Sustainability, Avenida Rovisco Pais, 1049-001 Lisbon, Portugal; 3 Centre for Ecology, Evolution and Environmental Changes (cE3c), Museu Nacional de História Natural e da Ciência, Rua da Escola Politécnica 56/58, 1250-102 Lisbon, Portugal

**Keywords:** Freshwater ecology, Freshwater ecology, History

## Abstract

The insights that historical evidence of human presence and man-made documents provide are unique. For example, using historical data may be critical to adequately understand the ecological requirements of species. However, historical information about freshwater species distribution remains largely a knowledge gap. In this Data Descriptor, we present the Portuguese Historical Fish Database (PHish–DB), a compilation of 2214 records (557 at the basin scale, 184 at the sub-basin scale and 1473 at the segment scale) resulting from a survey of 194 historical documents. The database was developed using a three-scale approach that maximises the inclusion of information by allowing different degrees of spatial acuity. PHish database contains records of 25 taxonomical groups and covers a time span of one millennium, from the 11th until the 20th century. This database has already proven useful for two scientific studies, and PHish further use will contribute to correctly assess the full range of conditions tolerated by species, by establishing adequate benchmark conditions, and/or to improve existing knowledge of the species distribution limits.

## Background & Summary

Collecting historical data on species diversity and occurrence from time periods earlier than the major impactful human activities taking place (e.g., damming, the Industrial Revolution, modern fishing, and river channelisation), may lead to an improvement of knowledge about species ecology. However, historical documents have limitations that need to be understood to avoid incorrect interpretations^1^ and/or extrapolations^2^. There is cultural filtering that affects not only the spatial and temporal availability, completeness, and reliability of documentary records but also their quantity and quality^2^. Most historical records rely factually on questionnaires/interviews, hence if there are erroneous answers, the inventories or scientific surveys will present incorrect data^3^. Nevertheless, the utility of historical insight cannot be underestimated and using historical data for ecological studies is valid^2^. Using an interdisciplinary approach^4^, combining information from several independent spatial and temporal sources^1,2,4,5^, cross-checking lines of evidence with independent datasets^6^ and blending different methods^2^ can help mitigate the limitations and lead to more accurate knowledge about past ecosystem conditions.

**Figure 1:**
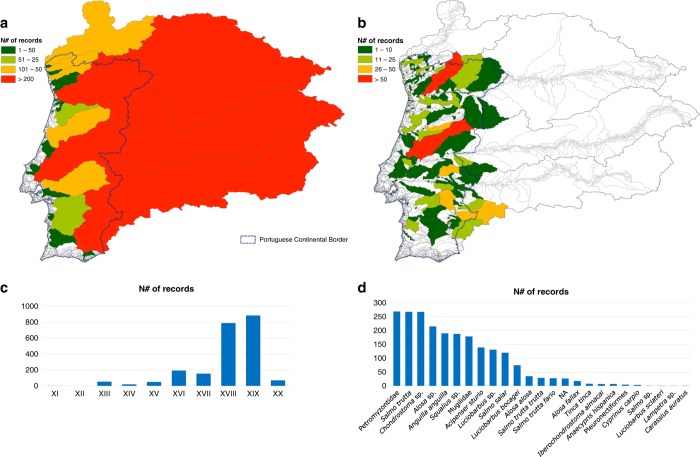
Summary results of the PHish database. (**a**)–Number of Records per river basin; (**b**)–Number of Records per river sub-basin; (**c**)–Number of Records per century; (**d**)–Number of Records per taxonomical group present in the field “Group Name”.

For some organisms and specific purposes, historical data might be essential to model the potential species distribution (e.g., Lassalle and Rochard^7^, Clavero and Hermoso^8^) because current distributions are often highly constrained by anthropogenic pressures that alter the natural realised ecological niche. A typical example is the case of diadromous fish species with their inland progression being gradually constrained by the presence of artificial barriers^8,9^. Consequently, current occurrence data will only cover a restricted range of the full conditions tolerated by species. To create the Portuguese Historical Fish Database (PHish–DB) we scouted 194 historical documents, resulting in 2214 records from 30 basins, 280 sub-basins and 490 segments. Data collection started in 2007 and was performed by researchers in history and ecology. Despite some underrepresentation of coastal areas, the spatial distribution of the historical records is homogeneous throughout the country and covers all the major river basins (Fig. 1). Three international river basins stand out (Douro, Minho and Guadiana) with a high number of records (Fig. 1a). The sub-basins with the highest number of records are from the River Tâmega (Douro) and River Zêzere (Tagus) (Fig. 1b). Spatial acuity of the records depended on the information present in the historical source. Thus, we opted for a three-scale approach to maximise the collected information. This has resulted in 557 records limited to the basin scale, 184 reaching the sub-basin scale, and 1473 records identified down to the highest accurate spatial scale, the river segment. PHish database covers a time span of one millennium, from the 11^th^ until the 20^th^ century, having a larger number of records for the second half of the millennium and particularly for the 18^th^ and 19^th^ centuries (Fig. 1c). The Interpretation of historical data can be very subjective^2^, and matching ancient fish common names with current taxonomy was challenging. To minimise uncertainty in the taxonomical classification of a fish record, a conservative approach was followed to establish the adequate taxonomical groups. Of the 25 group names defined from the records gathered, three stood out: Petromizontidae*, Chondrostoma* sp. and *Salmo trutta* (Fig. 1d).

The information present in the database has been partially used in the work of Segurado, *et al*.^10^, and also incorporated in a relevant European project, the European Fish Index–Plus (EFI + ) (http://efi-plus.boku.ac.at/). This database can nevertheless be useful to: improve existing scientific knowledge in Iberian context (e.g., Clavero and Hermoso^8^, Clavero, *et al*.^11^); expand scientific knowledge in European context via an Iberian occurrence scenario of a species with broad-European distribution (e.g., Filipe, *et al*.^12^); be used for research where historical interactions between human activities and riverine fish communities and population are relevant.

## Methods

These methods are expanded and updated versions of descriptions in our related work Segurado, *et al*.^10^.

### Historical sources

The present historical records compilation of riverine fish distribution was based on geographical dictionaries and other published information for Portugal, dated between the 11^th^ and early 20^th^ centuries. Portugal is the most south-western part of the European continent, representing 15% of the Iberian Peninsula. There are four major international rivers (Douro, Guadiana, Minho, and Tagus) and numerous other relevant national rivers. The available historical information on fish populations for this period was almost exclusively based on qualitative data of species occurrence. Available sources dated before the 16^th^ century included charters, inquiries, donations, and monastic chronicles. From the 16^th^ century onwards, more thorough recordings of the patrimony of the Portuguese kingdom were available, with the emergence of chorographies, historical-geographical memos, parish inquiries and dictionaries that recorded historically and geographically the Portuguese landscape. In addition to these sources, information from private libraries was also included. A total of 194 documents were consulted (Table 1 (available online only)). These historical sources contain information varying from aspects of the Portuguese physical territory, records about the natural resources of rivers, or cultural context of fisheries exploitation. Most of this data were compiled in the context of the EU projects EFI + (http://efi-plus.boku.ac.at/) and DURERO (Douro River Basin: Water Resources, Water Accounts and Target Sustainability Indices; http://138.100.137.130/durero_project_2014/), with the main purpose of providing data on reference conditions to compute biotic indicators based on diadromous species. Many regions of Europe have been shaped for centuries by human activities, leading to an absence of natural reference conditions for many water body types^13^. Hence, the definition of benchmark conditions may depend on the availability of historical sources of information on species occurrence^14^. This is especially relevant in the context of the Water Framework Directive of the European Union (WFD)^15^, which involves the assessment of the ecological quality of water bodies using the reference condition approach, in which quality classes are defined according to deviations from benchmark conditions.

### Taxonomical precision

Taxonomic acuity is critical to provide the best possible taxonomy insights from historical records and to derive reliable databases to be used as sources of information to test scientific or management hypotheses. However, this condition is rather challenging to attain when looking at large spatial scales. Indeed, in historical texts, the norm is to use local common names, mostly because many of the records predate the scientific description of the species. Therefore, the first step to produce this database was to establish a reference list by collecting and compiling ancient and current common names with their correspondence to scientific nomenclature. The second step was to attribute a valid species to each record, with an extra challenge when distinct common names are attributed to the same species among different regions. However, the most challenging issues are posed when several species share common names in certain regions or when very similar and even congener species are sympatric. Despite these caveats, because of the known present distribution of species, the reduced sympatry of similar species and the fact that most shared common names are of similar allopatric species, it is possible to attribute valid scientific identities to each record without errors. Whenever this attribution was impossible or uncertain, the genus, family or order was attributed to the record, instead of the species-specific epithet. This was the case for the genera *Alosa*, *Luciobarbus, Lampetra, Salmo* and *Squalius*, for the families Petromyzontidae and Mugilidae, and for order Pleuronectiformes. For the nases, it was decided to use an older genus’ name – *Chondrostoma*, valid in Europe, but with no current taxonomic validity in the Iberian Peninsula – that currently represents seven species in this database. This older genus aggregates three recently described genera (*Achondrostoma*, *Iberochondrostoma* and *Pseudochondrostoma*)^16^ that are, basin-wise, sympatric, coexisting at least two of these genera per basin with historical records. For Pleuronectiformes, the decision was made because it was unsure whether the species record corresponded to a freshwater species (*Plactichthys flesus*) or to a marine fish, of which there are several species. A conservative approach was followed and whenever a possibility for misinterpretation existed, the species were aggregated to the corresponding upper taxonomic level under the column “Group Name” (information that we recommend to use without any uncertainty). Whenever there were plausible reasons to believe that the record belonged to a given species, the full scientific binomial nomenclature was attributed to the column “Sub-group Name”. This has some associated uncertainty as the decision was made by expert judgement based on the available information. Whenever no plausibility existed, the higher taxonomic group (genus or family) was maintained without the attribution of a “Sub-group Name”. If there existed a possibility of confusion between species that did not fit the higher taxonomical groups defined, NA was attributed to “Group Name”. If plausible, an educated guess, for a species or a taxonomical group, was made into the “Sub-group Name”, based on the interpretation of the historical text extract. All the species and species groups considered are detailed in Table 2. To add value to the database, whenever available, information about the phenology and conservation status (national and international) was included.

### Georeferencing

To create a spatial representation of the historical data we have used the Catchment Characterisation and Modelling– River and Catchment database v2.1 (CCM2) (http://data.europa.eu/89h/fe1878e8-7541-4c66-8453-afdae7469221). An advantage of this pan-European database is its hierarchical structure, besides representing a fully integrated system between rivers and drainage catchments^17^. Using three spatial scales (basin, sub-basin and segment) allowed storing historical records with distinct spatial accuracy. Even though finer scales are more informative, historical data at a coarser scale is not irrelevant. For the basin scale, we used the identification code that CCM2 gives for each basin (WSO_ID) to link an historical record to this scale level. The same procedure was established at the segment scale, using the ID code that CCM2 assigns for each segment (WSO1_ID). Since CCM2 does not have any identification or spatial representation of the sub-basins within each sea outlet basin, we used a free software to create this information, the River Network Toolkit (RivTool). This software (available at www.rivtoolkit.com) uses integrated data about river networks and landscape/environmental datasets to produce new or aggregated data via calculations that consider the directional hierarchical network nature of rivers. The set of natural sub-basins of all sea outlet basins of the study area was created using the “sub-basin ID” function of RivTool.

The descriptions found in the historical sources varied greatly in their geographical precision. Most presence records referred to a given river or stream within a restricted region, usually described as being near a given village, township or city. When the geographic location was available, the record was georeferenced in a Geographical Information System (GIS) using CCM2. These were the most spatially precise records, the segment scale, where a segment corresponds to a river reach between two consecutive tributaries. In some cases, regions or town names were obsolete and further investigation was needed to clarify the current location and/or designation associated with that former nomination. Nevertheless, for some historical records, the former names did not have any information or relation with the current designations or did not have enough precision to be linked to a river segment. In those cases, the record was attributed to a higher spatial scale (Sub-basin or Basin). Data entries that could only be related to a watercourse that is a major river or stream that flows to the Atlantic Ocean, coastal lagoons or estuaries, were considered as low precision records and spatially defined at the basin scale. When the watercourse was identified as a tributary (i.e., smaller river or stream not flowing to the Ocean, coastal lagoon or estuary), the precision was considered higher and the record was spatially defined at the Sub-basin scale.

## Data Records

**Figure 2:**
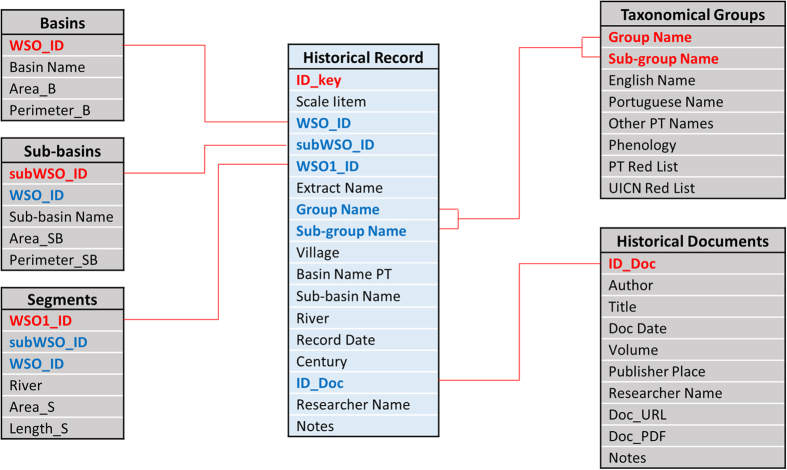
The relational structure of the Portuguese Historical Fish Database. Each box represents one table, with the header of a box indicating the name of the table, followed by the list of fields included in the table. The red lines indicate the primary relationship between tables; red fields are the primary key of each table; fields in blue indicate secondary relationships between tables.

A relational database structure (Fig. 2) was created in Microsoft Access® (available in the .accdb file extension) to adequately organise and store the historical data collected with their spatial and temporal dependencies, and also to maintain their link to the historical sources (Table 1 (available online only)). The PHish database is publicly available at the Open Science Framework (Data Citation 1) and at the University of Lisbon, School of Agriculture repository http://www.isa.ulisboa.pt/proj/PHish/. The database contains six tables: three related with spatial organisation, “Basins”, “Sub-basins”, “Segments”; one with taxonomical identification, “Taxonomical Groups”; one establishing the details of historical sources, “Historical Documents”; and finally, one aggregating historical record information with respective spatial, taxonomical and historical source information, “Historical Records” (Table 3 (available online only)). The latter table is the core of the relational database structure, relating to all other tables (Fig. 2) and where resulting historical data are stored (Table 3 (available online only)).

## Technical Validation

Interpretation of historical data can be very subjective and historical science is mostly inductive^2^. To increase objectivity and guarantee a correct assessment of historical information it is necessary to perform a critical evaluation of sources^3^, while comparing and combining multiple and independent sources and methods^18^. Special attention was taken to verify if authors were not just replicating information from other sources, and by that leading to duplication of results in the database. This was done not only while researchers were reading and surveying the historical documents and sources, but also by analysing, comparing and searching within the final set of historical records for similarities. For example, combined similarities in taxonomical groups and spatial references, similarities in paragraphs, sentences or parts of sentences were normally an indication that the author was just citing text from another document without acknowledging it explicitly. Whenever there was reasonable doubt about the originality of the information present in the historical source, or of the historical record, only the oldest one was included in the database.

Despite the numerous hurdles, taxonomical identification of ancient species common names followed a conservative approach that guaranteed no uncertainty for the “Group Name” field. Concerning the “Sub-group Name” field, the integration of information between spatial location and taxonomical identification of a record, the reliable considerations based on literature and the knowledge of experienced ecologists assured low levels of uncertainty. Moreover, when there was reasonable doubt or lack of plausibility, no consideration was made.

Spatial information for the records location was primarily accessed based on three Portuguese official water management and administrative sources at GIS environment: 1) Rivers map (Shapefile from www.hidrografico.pt); 2) Administrative regions and municipalities map (Shapefile from www.dgterritorio.pt); and 3) Online orthophotomaps (WMS link from www.igeo.pt). When the record city/council name or region was not easily connected to the information available in maps, numerous municipalities and parish websites were consulted, along with other websites from relevant local or regional associations, to help identify the more site-specific or out-dated spatial references. The connection with the CCM2 database was performed only after this thorough process. Records for places or historical locations which were not geographically identifiable were conservatively handled, either by discarding or including them in upper spatial scales (sub-basin or basin scale) when the river name was objectively identified.

## Usage Notes

Just like every database of historical records, the PHish database is neither definitive nor complete. All reasonable and possible updates will be held, though nevertheless dependent on resources and future funding. Methods will be maintained to avoid usage biases and/or interpretation issues. Future surveys for historical data should focus on rectifying spatial and temporal data heterogeneity. Obtaining information for older times (backward from the 16^th^ century) and focusing on the coastal areas of Portugal should bridge the spatial and temporal knowledge disparity.

Despite our best efforts, and even considering future updates to this database, true species occurrence will inevitably be broader than what historians and chroniclers may have reported. The cultural filtering^2^, accidental or intentional destruction of documents, doubtful sources of the historians/chroniclers and bias towards certain species^3^ affect both spatial and temporal availability, completeness, and reliability of documentary records^2^. In England, copyright law was limited to a special group of people until the 18^th^ century; indeed documents availability was still censored and limited to printers and publishers rights rather than to authors properties^19^. The concept of author’s intellectual property over its work only proliferated during the 19^th^ century, particularly in culturally developed countries such as France and Great Britain^20^. This means that at least until the 18^th^ century, and in Portugal most likely until the 19^th^ century, authors could quote other works without acknowledging them. Thus, data duplication is a possibility within this database, though we consider it in a very low probability given our cautious approach to this conundrum. Also, users must be aware that PHish database is a presence-only compilation of historical records. Without a wary systematic sampling, absence data is inevitably prone to a high degree of uncertainty, and to our best knowledge, no modern-day systematic survey of fish assemblages across the country was undertaken in Portugal until the end of the 20^th^ century.

The lack of data for coastal areas, and more specifically for the southern coastal regions of Portugal may result from several particular circumstances. Smaller basins are composed of smaller rivers and inevitably with less human settlements. Adding to this, southern Iberian rivers are Mediterranean-type freshwater ecosystems strongly shaped by autumn/winter flooding and summer drought events^21^. This seasonal instability has implications in the structure of freshwater communities^22^, meaning that these rivers will probably tend to support less interesting fishing areas and species. The database is also temporally unbalanced, i.e., although covering a vast time-scale it does not represent a consistent time-series due to lack or reduced number of records for the first half of the millennium. Future updates to this database will probably not fully overcome this as it is also the result of temporal filtering^3^ of historical sources. Another relevant issue is the heterogeneity of the established taxonomical groups in the user recommended field (Group Name). Our conservative approach followed herein avoids uncertainty in this classification, translating into correct and objective taxonomical information. However, for example, it may be thwarting to use some taxonomical groups (e.g., “Mugilidae” or *“Chondrostoma”)* when the objective is to perform species environmental niche modelling.

All mentioned issues, biases and unbalances are normal for historical databases, and none of them hampers the usage of the database. To our knowledge, this database is the first public compilation of historical distribution data based on freshwater fish species in Portuguese rivers and South-western Europe. Notwithstanding, users should keep in mind all these features and caveats whenever making any considerations or extrapolation based on this database. The PHish database is geographically limited since it is restricted to inland Portugal. However, the current database contains valuable information by covering data for the Portuguese areas, including sea outlets, of four Iberian international rivers and most Portuguese major watercourses. Moreover, because Portuguese data were not compiled and made available until now, researchers have been using only Spanish records to study fish species distribution for the whole Iberian Peninsula, minimizing its importance as a meaningful biogeographical entity^23^. By using only Spanish data, authors concede to uncertain premises (e.g., Clavero and Villero^24^) and/or extrapolate when predicting for the entire Iberian Peninsula (e.g., Clavero and Hermoso^8^). Hence, this database will fill an important gap in the current knowledge and contribute to the development of new studies covering the whole Iberian Peninsula without being hindered by political borders.

## Additional information

**How to cite this article**: Duarte, G. *et al*. One millennium of historical freshwater fish occurrence data for Portuguese rivers and streams. *Sci. Data* 5:180163 doi: 10.1038/sdata.2018.163 (2018).

**Publisher’s note**: Springer Nature remains neutral with regard to jurisdictional claims in published maps and institutional affiliations.

## Supplementary Material



## Figures and Tables

**Table 1:** List of historical sources surveyed to create the PHish database.

ID_Doc	Authors	Title	Doc_date	Volume	Publisher place
Almaça-Elvira_2000	Almaça C. & Elvira B.	Past and present distribution of *Acipenser sturio* L., 1758 on the Iberian Peninsula	2000	NA	Boletín. Instituto Español de Oceanografía, 16 (1-4): 11-16
Almeida_1866a	José Avelino d'Almeida	Dicionário abreviado de Corografia, Topografia e Arqueologia das Cidades, Vilas e Aldeias de Portugal (3 vol.)	1866a	1	Typographia de V. de Moraes, Valença
Almeida_1866b	José Avelino d'Almeida	Dicionário abreviado de Corografia, Topografia e Arqueologia das Cidades, Vilas e Aldeias de Portugal (3 vol.)	1866b	2	Typographia de V. de Moraes, Valença
Almeida_1866c	José Avelino d'Almeida	Dicionário abreviado de Corografia, Topografia e Arqueologia das Cidades, Vilas e Aldeias de Portugal (3 vol.)	1866c	3	Typographia de V. de Moraes, Valença
Amado_1861	José de Sousa Amado	Compendio de chorographia de Portugal. Seguido de Cartas chorographicas do reino e dos archipelagos dos Açores e da Madeira	1861	NA	Typographia de G. M. Martins, Lisboa
Amado_1874	José de Sousa Amado	Corografia da Lusitânia acompanhada de uma Carta Corográfica para uso dos alunos do 2º ano de Geografia e principalmente no exame final da disciplina	1874	NA	Typographia Universal, Lisboa
Andrade_1878a1	Agostinho Rodrigues de Andrade	Diccionario chorographico do reino de Portugal... seguido de dois pequenos diccionarios hydrographico e orographico do nosso paiz…	1878a1	NA	Imprensa da Universidade, Coimbra
Andrade_1878a2	Agostinho Rodrigues de Andrade	Pequeno Diccionario Hydrographico de Portugal	1878a2	NA	Imprensa da Universidade, Coimbra
Andrade_1944	António Sampaio de Andrade	Dicionário corográfico de Portugal contemporâneo: (Continente, Ilhas Adjacentes e Colónias), elaborado de acordo com as actuais divisões	1944	NA	Livraria Figueirinhas, Porto
Anónimo_1817	Anónimo	Descripção de Portugal	1817	NA	-
Aranha_1871	Pedro Wenceslau de Brito Aranha	Memórias Histórico-Estatísticas de Algumas Villas e Povoações de Portugal	1871	NA	Livraria de A. M. Pereira – Editor, Lisboa
ARCL_1784	Academia Real das Ciências de Lisboa	Memorias económicas da academia real das sciencias de Lisboa, para o adiantamento da agricultura, das artes, e da industria em Portugal, e suas conquistas	1784	1	Na Officina da Academia Real das Sciencias, Lisboa
ARCL_1790	Academia Real das Ciências de Lisboa	Memorias económicas da academia real das sciencias de Lisboa, para o adiantamento da agricultura, das artes, e da industria em Portugal, e suas conquistas	1790	2	Na Officina da Academia Real das Sciencias, Lisboa
ARCL_1791	Academia Real das Ciências de Lisboa	Memorias económicas da academia real das sciencias de Lisboa, para o adiantamento da agricultura, das artes, e da industria em Portugal, e suas conquistas	1791	3	Na Officina da Academia Real das Sciencias, Lisboa
Azevedo_1906	Francisco Cardoso de Azevedo	Novo diccionario chorographico de Portugal Continental e Insular contendo as divisões administrativa, judicial, ecclesiastica e militar [...]	1906	NA	Typ. a Vapor de José da Silva Mendonça, Porto
Azevedo_1997	José Correia de Azevedo	Por terras e rios portugueses de trutas	1997	NA	Edições J. Correia, Braga
Baptista_1875a	João Maria Baptista	Chorographia Moderna do reino de Portugal (7 vol.)	1875a	2	Typographia da Academia real das Sciencias, Lisboa
Baptista_1875b	João Maria Baptista	Chorographia Moderna do reino de Portugal (7 vol.)	1875b	3	Typographia da Academia real das Sciencias, Lisboa
Baptista_1876a	João Maria Baptista	Chorographia Moderna do reino de Portugal (7 vol.)	1876a	4	Typographia da Academia real das Sciencias, Lisboa
Baptista_1876b	João Maria Baptista	Chorographia Moderna do reino de Portugal (7 vol.)	1876b	5	Typographia da Academia real das Sciencias, Lisboa
Barbosa_1860a	Ignacio de Vilhena Barbosa	As cidades e villas da monarchia portugueza que teem brasão d'armas	1860a	1	Typographia do Panorama, Lisboa
Barbosa_1860b	Ignacio de Vilhena Barbosa	As cidades e villas da monarchia portugueza que teem brasão d'armas	1860b	2	Typographia do Panorama, Lisboa
Barbosa_1862	Ignacio de Vilhena Barbosa	As cidades e villas da monarchia portugueza que teem brasão d'armas	1862	3	Typographia do Panorama, Lisboa
Basto_1980	Artur de Magalhães Basto	Vereaçoens : anos de 1390-1395 : o mais antigo dos livros de Vereaçoes do Municipio do Porto existentes no seu Arquivo	1980	NA	Publicações da Câmara Municipal do Porto, Porto
Bastos_2006	Maria Rosário da Costa Bastos	O baixo Vouga em tempos medievos: do preâmbulo da Monarquia aos finais do reinado de D.Dinis	2006	NA	Tese de Doutoramento em Ciências Humanas e Sociais na especialidade de História apresentada à Universidade Aberta, Lisboa
Bettencourt_1874	Emiliano Augusto de Bettencourt	Diccionario chorographico de Portugal com as divisões administrativa, judicial, ecclesiastica e militar... precedido de um resumo de chorographia patria	1874	NA	Typographia Sousa & Filho, Lisboa
Bettencourt_1889	Emiliano Augusto de Bettencourt	Noções de chorographia de Portugal: seguidas da carta geographica do continente, e de um planispherio onde se indica a posição geographica das possessões portuguezas	1889	NA	A. Ferreira Machado & Cª, Lisboa
Bezerra_1785	Manuel Gomes de Lima Bezerra	Os estrangeiros no Lima, ou Conversaçoens eruditas sobre os varios pontos de historia ecclesiastica, civil, litteraria, natural, genealogica, antiguidades, geographia, agricultura, commercio, artes, e sciencias. Com huma descripção de todas a[...]	1785	NA	Real Officina da Universidade, Coimbra
Brito_1597	O. Cist. Bernardo de Brito	Geografia Antiga de Lusytania	1597	NA	António Alvarez, Impressor de Livros, Alcobaça
Cabreira-Cabral_1942	Estefânia Cabreira & Oliveira Cabral	Corografia de Portugal e seu Império	1942	NA	Editorial Domingos Barreira
Câmara_1850	Paulo Perestrello da Câmara	Diccionário geographico, histórico, político e litterário do reino de Portugal e seus domínios	1850	1	Typographia Universal E. e H. Laemmert, Rio de Janeiro
Cardoso_1832	Luís Cardoso (Ed.)	Memórias Paroquiais	1832	1	Arquivo Nacional da Torre do Tombo
Cardoso_1832a	Luís Cardoso (Ed.)	Memórias Paroquiais	1832a	2	Arquivo Nacional da Torre do Tombo
Cardoso_1832aa	Luís Cardoso (Ed.)	Memórias Paroquiais	1832aa	33	Arquivo Nacional da Torre do Tombo
Cardoso_1832ab	Luís Cardoso (Ed.)	Memórias Paroquiais	1832ab	34	Arquivo Nacional da Torre do Tombo
Cardoso_1832ac	Luís Cardoso (Ed.)	Memórias Paroquiais	1832ac	35	Arquivo Nacional da Torre do Tombo
Cardoso_1832ad	Luís Cardoso (Ed.)	Memórias Paroquiais	1832ad	36	Arquivo Nacional da Torre do Tombo
Cardoso_1832ae	Luís Cardoso (Ed.)	Memórias Paroquiais	1832ae	38	Arquivo Nacional da Torre do Tombo
Cardoso_1832af	Luís Cardoso (Ed.)	Memórias Paroquiais	1832af	39	Arquivo Nacional da Torre do Tombo
Cardoso_1832ag	Luís Cardoso (Ed.)	Memórias Paroquiais	1832ag	41	Arquivo Nacional da Torre do Tombo
Cardoso_1832b	Luís Cardoso (Ed.)	Memórias Paroquiais	1832b	3	Arquivo Nacional da Torre do Tombo
Cardoso_1832c	Luís Cardoso (Ed.)	Memórias Paroquiais	1832c	4	Arquivo Nacional da Torre do Tombo
Cardoso_1832d	Luís Cardoso (Ed.)	Memórias Paroquiais	1832d	5	Arquivo Nacional da Torre do Tombo
Cardoso_1832e	Luís Cardoso (Ed.)	Memórias Paroquiais	1832e	6	Arquivo Nacional da Torre do Tombo
Cardoso_1832f	Luís Cardoso (Ed.)	Memórias Paroquiais	1832f	7	Arquivo Nacional da Torre do Tombo
Cardoso_1832g	Luís Cardoso (Ed.)	Memórias Paroquiais	1832g	8	Arquivo Nacional da Torre do Tombo
Cardoso_1832h	Luís Cardoso (Ed.)	Memórias Paroquiais	1832h	10	Arquivo Nacional da Torre do Tombo
Cardoso_1832i	Luís Cardoso (Ed.)	Memórias Paroquiais	1832i	11	Arquivo Nacional da Torre do Tombo
Cardoso_1832j	Luís Cardoso (Ed.)	Memórias Paroquiais	1832j	12	Arquivo Nacional da Torre do Tombo
Cardoso_1832k	Luís Cardoso (Ed.)	Memórias Paroquiais	1832k	13	Arquivo Nacional da Torre do Tombo
Cardoso_1832l	Luís Cardoso (Ed.)	Memórias Paroquiais	1832l	14	Arquivo Nacional da Torre do Tombo
Cardoso_1832m	Luís Cardoso (Ed.)	Memórias Paroquiais	1832m	15	Arquivo Nacional da Torre do Tombo
Cardoso_1832n	Luís Cardoso (Ed.)	Memórias Paroquiais	1832n	16	Arquivo Nacional da Torre do Tombo
Cardoso_1832o	Luís Cardoso (Ed.)	Memórias Paroquiais	1832o	18	Arquivo Nacional da Torre do Tombo
Cardoso_1832p	Luís Cardoso (Ed.)	Memórias Paroquiais	1832p	20	Arquivo Nacional da Torre do Tombo
Cardoso_1832q	Luís Cardoso (Ed.)	Memórias Paroquiais	1832q	21	Arquivo Nacional da Torre do Tombo
Cardoso_1832r	Luís Cardoso (Ed.)	Memórias Paroquiais	1832r	22	Arquivo Nacional da Torre do Tombo
Cardoso_1832s	Luís Cardoso (Ed.)	Memórias Paroquiais	1832s	23	Arquivo Nacional da Torre do Tombo
Cardoso_1832t	Luís Cardoso (Ed.)	Memórias Paroquiais	1832t	24	Arquivo Nacional da Torre do Tombo
Cardoso_1832u	Luís Cardoso (Ed.)	Memórias Paroquiais	1832u	25	Arquivo Nacional da Torre do Tombo
Cardoso_1832v	Luís Cardoso (Ed.)	Memórias Paroquiais	1832v	26	Arquivo Nacional da Torre do Tombo
Cardoso_1832w	Luís Cardoso (Ed.)	Memórias Paroquiais	1832w	27	Arquivo Nacional da Torre do Tombo
Cardoso_1832x	Luís Cardoso (Ed.)	Memórias Paroquiais	1832x	28	Arquivo Nacional da Torre do Tombo
Cardoso_1832y	Luís Cardoso (Ed.)	Memórias Paroquiais	1832y	29	Arquivo Nacional da Torre do Tombo
Cardoso_1832z	Luís Cardoso (Ed.)	Memórias Paroquiais	1832z	32	Arquivo Nacional da Torre do Tombo
Carvalho_1765	José Monteiro de Carvalho	Diccionario Portuguez das Plantas, Arbustos, […]	1765	NA	Oficina de Miguel Manescal da Costa, Impressor do Santo Officio, Lisboa
Carvalho_1987	Rómulo de Carvalho	A História Natural em Portugal no Século XVIII	1987	NA	Oficinas Gráficas da Minerva do Comércio, Lisboa
Castro_1762	João Baptista de Castro	Mappa de Portugal Antigo e Moderno	1762	1	Officina Patriarcal de Francisco Luiz Ameno, Lisboa
Castro_1815	Lourenço da Mesquita Pimentel Sotto-Maior e Castr	Mappa Chronologico do reino de Portugal	1815	NA	Impressão de J. B. Morando, Lisboa
Castro_1966	Armando Castro	A evolução económica de Portugal dos séculos XII a XV	1966	4	Editora Portugália, Lisboa
Coelho_1990	Maria Helena da Cruz Coelho	Apontamentos sobre a comida e a bebida do campesinato coimbrão em tempos medievos. In: Homens, espaços e poderes (séculos XI a XVI)	1990	NA	Notas do viver social. Lisboa: Horizonte, 1990, p. 9-22
Coelho_1995	Maria Helena da Cruz Coelho	A pesca fluvial na economia e na sociedade medieval portuguesa	1995	NA	FLUC Secção de História - Artigos em Livros de Actas, Lisboa
Coroa_1258	Feitos da Coroa	Livro 5 de Inquirições de Dom Afonso III	1258	NA	Arquivo Nacional Torre do Tombo (PT/TT/FC/2/8)
Costa_1706	António Carvalho da Costa	Corografia Portuguesa e Descripçam Topográfica do famoso reyno de Portugal (3vol.)	1706	1	Officina de Valentim da Costa Deslandes impressor de Sua Magestade, & á sua custa impresso, Lisboa
Costa_1708	António Carvalho da Costa	Corografia Portuguesa e Descripçam Topográfica do famoso reyno de Portugal (3vol.)	1708	2	Officina de Valentim da Costa Deslandes impressor de Sua Magestade, & á sua custa impresso, Lisboa
Costa_1712	António Carvalho da Costa	Corografia Portuguesa e Descripçam Topográfica do famoso reyno de Portugal (3vol.)	1712	3	Oficina Real Deslandesiana, Lisboa
Costa_1789	Agostinho Rebelo da Costa	Descripção Topographica e Historica da cidade do Porto. Que contém a sua origem, situaçaõ, e antiguidades: a magnificencia dos seus templos, mosteiros, hospitaes, ruas, praças, edificios, e fontes...	1789	NA	Na Officina de Antonio Alvarez Ribeiro, Porto
Costa_1929	Américo Costa	Diccionario Chorographico de Portugal Continental e Insular: Hydrographico, Historico, Orographico, Biographico, Archeologico, Heraldico, Etymologico	1929	1	Livraria Civilização, Porto
Costa_1930	Américo Costa	Diccionario Chorographico de Portugal Continental e Insular: Hydrographico, Historico, Orographico, Biographico, Archeologico, Heraldico, Etymologico	1930	2	Livraria Civilização, Porto
Costa_1932	Américo Costa	Diccionario Chorographico de Portugal Continental e Insular: Hydrographico, Historico, Orographico, Biographico, Archeologico, Heraldico, Etymologico	1932	3	Livraria Civilização, Porto
Costa_1934	Américo Costa	Diccionario Chorographico de Portugal Continental e Insular: Hydrographico, Historico, Orographico, Biographico, Archeologico, Heraldico, Etymologico	1934	4	Livraria Civilização, Porto
Costa_1936	Américo Costa	Diccionario Chorographico de Portugal Continental e Insular: Hydrographico, Historico, Orographico, Biographico, Archeologico, Heraldico, Etymologico	1936	5	Livraria Civilização, Porto
Costa_1938	Américo Costa	Diccionario Chorographico de Portugal Continental e Insular: Hydrographico, Historico, Orographico, Biographico, Archeologico, Heraldico, Etymologico	1938	6	Livraria Civilização, Porto
Costa_1940	Américo Costa	Diccionario Chorographico de Portugal Continental e Insular: Hydrographico, Historico, Orographico, Biographico, Archeologico, Heraldico, Etymologico	1940	7	Livraria Civilização, Porto
Costa_1943	Américo Costa	Diccionario Chorographico de Portugal Continental e Insular: Hydrographico, Historico, Orographico, Biographico, Archeologico, Heraldico, Etymologico	1943	8	Livraria Civilização, Porto
Costa_1947	Américo Costa	Diccionario Chorographico de Portugal Continental e Insular: Hydrographico, Historico, Orographico, Biographico, Archeologico, Heraldico, Etymologico	1947	9	Livraria Civilização, Porto
Costa_1948a	Américo Costa	Diccionario Chorographico de Portugal Continental e Insular: Hydrographico, Historico, Orographico, Biographico, Archeologico, Heraldico, Etymologico	1948a	10	Livraria Civilização, Porto
Costa_1948b	Américo Costa	Diccionario Chorographico de Portugal Continental e Insular: Hydrographico, Historico, Orographico, Biographico, Archeologico, Heraldico, Etymologico	1948b	11	Livraria Civilização, Porto
Costa_1949	Américo Costa	Diccionario Chorographico de Portugal Continental e Insular: Hydrographico, Historico, Orographico, Biographico, Archeologico, Heraldico, Etymologico	1949	12	Livraria Civilização, Porto
Denis_1846a	M. Fernando Denis	Portugal pittoresco ou Descripção historica d'este reino	1846a	1	Typografia de L.C. da Cunha, Lisboa
Denis_1846b	M. Fernando Denis	Portugal pittoresco ou Descripção historica d'este reino	1846b	2	Uma Sociedade, Typografia de L.C. da Cunha, Lisboa
Denis_1847	M. Fernando Denis	Portugal pittoresco ou Descripção historica d'este reino	1847	3	Uma Sociedade, Typografia de L.C. da Cunha, Lisboa
Denis_1849	M. Fernando Denis	Portugal pittoresco, ou, Descripção historica d'este reino	1849	4	Uma Sociedade, Typografia de L.C. da Cunha, Lisboa
Deusdado_1893	Manuel António Ferreira Deusdado	Chorographia de Portugal illustrada [com] 50 gravuras [e] 20 mappas a cores	1893	NA	Guillard, Aillaud & Cª, Lisboa
DGSFA_1965	Direcção-Geral dos Serviços Florestais e Aquícolas	Portaria nº21295 - Aprova o Regulamento Especial para a Zona de Pesca da Lagoa Comprida	1965	NA	Ministério da Economia, Lisboa
DGSFA_1966	Direcção-Geral dos Serviços Florestais e Aquícolas	Portaria nº22040 - Aprova o Regulamento Especial para a Zona de Pesca Reservada que se designa por «Grupo das pequenas lagoas da Serra da Estrela»	1966	NA	Ministério da Economia, Lisboa
Dinis_1960	Dias Dinis	Estudos Henriquinos	1960	1	Acta Universitatis Conimbrigensis, Coimbra
DPAI_1999	Direcção-Geral das Florestas (DGF) – Divisão de Pe	Gestão dos recursos aquícolas em Portugal	1999	NA	Direcção-Geral das Florestas, Lisboa
Eça_1877	Bento Fortunato de Moura Coutinho de Almeida d'Eça	Memorias ácerca do regimen do Tejo e outros rios	1877	NA	Imprensa Nacional, Lisboa
Faria_1655	Manuel Severim de Faria	Noticias de Portugal: offerecidas a El Rey N.S. Dom João o IV	1655	NA	Na Officina Craesbeeckiana, Lisboa
Fernandes_1937	Manuel Fernandes	Memorial da corografia de Portugal	1937	2	-
Ferreira_1608	José Martins Ferreira	Breve Compendio, ou summario das grandezas, e cousas notaveis da comarca entre Douro, e Minho, com a lista dos condestaveis de Portugal, e Vice-Reis da India	1608	NA	-
Figueiredo-Lima_1817	Manuel de Figueiredo & Henrique de Campos Ferreira	Descripção de Portugal : apontamentos e notas da sua historia antiga, e moderna, ecclesiastica, civil, e militar	1817	NA	Tipografia Lacerdina, Lisboa
Franclim_1999	Francisco Nunes Franclim	Terras portuguesas: arquivo histórico-corográfico ou corografia histórica portuguesa	1999	NA	Oficinas gráficas Copi-Pronto; EDINOVA, Lisboa
Frazão_1952	António César Amaral Frazão	Novo Dicionário Corográfico de Portugal (Continente, Ilhas Adjacentes e Colónias)	1952	NA	Domingos Barreira, Porto
Frazão_1981	António César Amaral Frazão	Novo dicionário corográfico de Portugal: Continente e Ilhas Adjacentes	1981	NA	Domingos Barreira, Porto
Freire_1739	António de Oliveira Freire	Descripçam Corografica do reyno de Portugal que contem huma exacta relaçam de suas Provincias, Comarcas, Cidades, Villas, Freguezias, montes, rios, portos […]	1739	NA	Officina de Miguel Rodrigues, Lisboa
Freire_1870	Henrique Augusto da Cunha Soares Freire	Compendio de chorographia de Portugal	1870	-	-
Frias_1886	David Correia Sanches de Frias	Notas a lápis, passeios e digressões peninsulares	1886	NA	Edição António Maria Pereira, Lisboa
Geraldes_1999	Ana Maria Geraldes	Peixes de água doce	1999	NA	João Azevedo Editores, Mirandela
Gomes_2011	Sandra Rute Fonseca Gomes	Territórios Medievais do Pescado do Reino de Portugal	2011	NA	Dissertação de mestrado em Alimentação - Fontes, Cultura e Sociedade, apresentada à Faculdade de Letras da Universidade de Coimbra
Gonçalves_2012	Iria Gonçalves	Por terras de entre-Douro-e-Minho com as inquirições de Dom Afonso III	2012	NA	Edições Afrontamento Lda., Porto
Guerra_1861	Manuel José Júlio Guerra	Estudos chorographicos, phisicos e hidrographicos da bacia do Tejo comprehendida no Reino de Portugal	1861	NA	Imprensa Nacional, Lisboa
HFAC_1980	História Florestal, Aquícola e Cinegética	Colectânea de Documentos existentes no Arquivo Nacional da Torre do Tombo. Chancelarias Reais (1208-1483), direcção e selecção de C.M.L. Baeta Neves, transcrição e revisão de provas de Maria Teresa Barbosa Acabado, compilação, sumários e índices de Maria	1980	1	Ministério da Agricultura e Pescas, Direcção-Geral do Ordenamento e Gestão Florestal
HFAC_1982a	História Florestal, Aquícola e Cinegética	Colectânea de Documentos existentes no Arquivo Nacional da Torre do Tombo. Chancelarias Reais (1439-1481), direcção e selecção de C.M.L. Baeta Neves, transcrição e revisão de provas de Maria Teresa Barbosa Acabado, compilação, sumários e índices de Maria	1982a	2	Ministério da Agricultura e Pescas, Direcção-Geral do Ordenamento e Gestão Florestal
HFAC_1982b	História Florestal, Aquícola e Cinegética	Colectânea de Documentos existentes no Arquivo Nacional da Torre do Tombo. Chancelarias Reais (1481-1493), direcção e selecção de C.M.L. Baeta Neves, transcrição e revisão de provas de Maria Teresa Barbosa Acabado, compilação, sumários e índices de Maria	1982b	3	Ministério da Agricultura e Pescas, Direcção-Geral do Ordenamento e Gestão Florestal
HFAC_1983	História Florestal, Aquícola e Cinegética	Colectânea de Documentos existentes no Arquivo Nacional da Torre do Tombo. Chancelarias Reais (1495-1521), direcção e selecção de C.M.L. Baeta Neves, transcrição e revisão de provas de Maria Teresa Barbosa Acabado, compilação, sumários e índices de Maria	1983	4	Ministério da Agricultura e Pescas, Direcção-Geral do Ordenamento e Gestão Florestal
HFAC_1988	História Florestal, Aquícola e Cinegética	Colectânea de Documentos existentes no Arquivo Nacional da Torre do Tombo. Chancelarias Reais (1521-1527), direcção e selecção de C.M.L. Baeta Neves, transcrição e revisão de provas de Maria Teresa Barbosa Acabado, compilação, sumários e índices de Maria	1988	5.1	Ministério da Agricultura e Pescas, Direcção-Geral do Ordenamento e Gestão Florestal
HFAC_1990	História Florestal, Aquícola e Cinegética	Colectânea de Documentos existentes no Arquivo Nacional da Torre do Tombo. Chancelarias Reais (1528-1564), direcção e selecção de C.M.L. Baeta Neves, transcrição e revisão de provas de Maria Teresa Barbosa Acabado, compilação, sumários e índices de Maria	1990	5.2	Ministério da Agricultura e Pescas, Direcção-Geral do Ordenamento e Gestão Florestal
HFAC_1993	História Florestal, Aquícola e Cinegética	Colectânea de Documentos existentes no Arquivo Nacional da Torre do Tombo. Chancelarias Reais (1553-1583), direcção e selecção de C.M.L. Baeta Neves, transcrição e revisão de provas de Maria Teresa Barbosa Acabado, compilação, sumários e índices de Maria	1993	6	Ministério da Agricultura e Pescas, Direcção-Geral do Ordenamento e Gestão Florestal
Ilharco_1947	João Ilharco	Corografia de Portugal e do império colonial português	1947	NA	Domingos Barreira, Porto
Júnior_1968	José Bragança Gil Júnior	Dicionário corográfico administrativo e judicial: Portugal Continental e Insular	1968	NA	Editoria Portugália, Lisboa
Lage_1897	José Gonçalves Lage	Elementos de chronologia, de geographia e de corographia de Portugal	1897	NA	-
Leal_1873	Augusto Soares de Azevedo Barbosa Pinho Leal	Portugal Antigo e Moderno - Diccionário Geográphico, Estatístico, Chorográphico; Heráldico, Archeologico; Histórico, Biographico e Etymologico de todas as Cidades, Villas e Freguezias de Portugal e grande número de Aldeias (12 vol.)	1873	1	Livraria Editora de Mattos Moreira & Companhia, Lisboa
Leal_1874a	Augusto Soares de Azevedo Barbosa Pinho Leal	Portugal Antigo e Moderno - Diccionário Geográphico, Estatístico, Chorográphico; Heráldico, Archeologico; Histórico, Biographico e Etymologico de todas as Cidades, Villas e Freguezias de Portugal e grande número de Aldeias (12 vol.)	1874a	2	Livraria Editora de Mattos Moreira & Companhia, Lisboa
Leal_1874b	Augusto Soares de Azevedo Barbosa Pinho Leal	Portugal Antigo e Moderno - Diccionário Geográphico, Estatístico, Chorográphico; Heráldico, Archeologico; Histórico, Biographico e Etymologico de todas as Cidades, Villas e Freguezias de Portugal e grande número de Aldeias (12 vol.)	1874b	3	Livraria Editora de Mattos Moreira & Companhia, Lisboa
Leal_1874c	Augusto Soares de Azevedo Barbosa Pinho Leal	Portugal Antigo e Moderno - Diccionário Geográphico, Estatístico, Chorográphico; Heráldico, Archeologico; Histórico, Biographico e Etymologico de todas as Cidades, Villas e Freguezias de Portugal e grande número de Aldeias (12 vol.)	1874c	4	Livraria Editora de Mattos Moreira & Companhia, Lisboa
Leal_1875a	Augusto Soares de Azevedo Barbosa Pinho Leal	Portugal Antigo e Moderno - Diccionário Geográphico, Estatístico, Chorográphico; Heráldico, Archeologico; Histórico, Biographico e Etymologico de todas as Cidades, Villas e Freguezias de Portugal e grande número de Aldeias (12 vol.)	1875a	5	Livraria Editora de Mattos Moreira & Companhia, Lisboa
Leal_1875b	Augusto Soares de Azevedo Barbosa Pinho Leal	Portugal Antigo e Moderno - Diccionário Geográphico, Estatístico, Chorográphico; Heráldico, Archeologico; Histórico, Biographico e Etymologico de todas as Cidades, Villas e Freguezias de Portugal e grande número de Aldeias (12 vol.)	1875b	6	Livraria Editora de Mattos Moreira & Companhia, Lisboa
Leal_1876	Augusto Soares de Azevedo Barbosa Pinho Leal	Portugal Antigo e Moderno - Diccionário Geográphico, Estatístico, Chorográphico; Heráldico, Archeologico; Histórico, Biographico e Etymologico de todas as Cidades, Villas e Freguezias de Portugal e grande número de Aldeias (12 vol.)	1876	7	Livraria Editora de Mattos Moreira & Companhia, Lisboa
Leal_1878	Augusto Soares de Azevedo Barbosa Pinho Leal	Portugal Antigo e Moderno - Diccionário Geográphico, Estatístico, Chorográphico; Heráldico, Archeologico; Histórico, Biographico e Etymologico de todas as Cidades, Villas e Freguezias de Portugal e grande número de Aldeias (12 vol.)	1878	8	Livraria Editora de Mattos Moreira & Companhia, Lisboa
Leal_1880	Augusto Soares de Azevedo Barbosa Pinho Leal	Portugal Antigo e Moderno - Diccionário Geográphico, Estatístico, Chorográphico; Heráldico, Archeologico; Histórico, Biographico e Etymologico de todas as Cidades, Villas e Freguezias de Portugal e grande número de Aldeias (12 vol.)	1880	9	Livraria Editora de Mattos Moreira & Companhia, Lisboa
Leal_1882	Augusto Soares de Azevedo Barbosa Pinho Leal	Portugal Antigo e Moderno - Diccionário Geográphico, Estatístico, Chorográphico; Heráldico, Archeologico; Histórico, Biographico e Etymologico de todas as Cidades, Villas e Freguezias de Portugal e grande número de Aldeias (12 vol.)	1882	10	Livraria Editora de Mattos Moreira, Lisboa
Leal_1886	Augusto Soares de Azevedo Barbosa Pinho Leal	Portugal Antigo e Moderno - Diccionário Geográphico, Estatístico, Chorográphico; Heráldico, Archeologico; Histórico, Biographico e Etymologico de todas as Cidades, Villas e Freguezias de Portugal e grande número de Aldeias (12 vol.)	1886	11	Livraria Editora de Tavares Cardoso & Irmão, Lisboa
Leal_1890	Augusto Soares de Azevedo Barbosa Pinho Leal	Portugal Antigo e Moderno - Diccionário Geográphico, Estatístico, Chorográphico; Heráldico, Archeologico; Histórico, Biographico e Etymologico de todas as Cidades, Villas e Freguezias de Portugal e grande número de Aldeias (12 vol.)	1890	12	Livraria Editora de Tavares Cardoso & Irmão, Lisboa
Leão_1610	Duarte Nunez do Leão	Descripção do reino de Portugal	1610	NA	Jorge Rodriguez, Lisboa
Lima_1935	Baptista de Lima	Terras Portuguesas: arquivo histórico-corográfico ou corografia histórica portuguesa	1935	3	Camões, Póvoa-de-Varzim
Lopes_1841	João Baptista da Silva Lopes	Corografia, ou Memoria economica, estadistica, e topografica do reino do Algarve	1841	NA	Typographia da Academia das Sciências de Lisboa, Lisboa
Lopes_1891	João Baptista da Silva Lopes	Diccionario postal e chorographico do Reino de Portugal comprehendendo a divisão administrativa, judicial e ecclesiastica do Continente do Reino e dos archipelagos dos Açores e Madeira	1891	1	Imprensa Nacional, Lisboa
Lopes_1893	João Baptista da Silva Lopes	Diccionario postal e chorographico do Reino de Portugal comprehendendo a divisão administrativa, judicial e ecclesiastica do Continente do Reino e dos archipelagos dos Açores e Madeira	1893	2	Imprensa Nacional, Lisboa
Lopes_1894	João Baptista da Silva Lopes	Diccionario postal e chorographico do Reino de Portugal comprehendendo a divisão administrativa, judicial e ecclesiastica do Continente do Reino e dos archipelagos dos Açores e Madeira	1894	3	Imprensa Nacional, Lisboa
Loureiro_1882	Adolfo Ferreira de Loureiro	Memória sobre o porto e a barra da Figueira e as obras para o seu melhoramento	1882	NA	Imprensa Nacional, Lisboa
Machado_1980	Fernando Falcão Machado	Corografia lusíada	1980	-	-
Maranhão_1852	Francisco dos Prazeres Maranhão	Diccionario geographico abreviado de Portugal e suas possessões ultramarinas […]	1852	NA	Typographia de Sebastião José Pereira, Porto
Marnay_1883	C. Marnay	Projecto do porto e melhoramento da barra do Douro, do Lima e do Mondego: considerações geraes, apreciações e descripção summaria d’este projecto com a demonstração da exequividade e das vantagens do systema adopta do e r	1883	NA	Typographia Occidental, Porto
Marques_1992	A. H. de Oliveira Marques (ccord.)	Chancelarias Portuguesas : D. Afonso IV, vol. II, 1336-1340	1992	2	Instituto Nacional de Investigação Científica / Centro de Estudos Históricos da Universidade Nova de Lisboa, Lisboa
Marques-Iria_1944	João Martins da Silva Marques & Alberto Iria	Descobrimentos Portugueses: Documentos para a sua História (1147-1460).	1944	1	Instituto para a Alta Cultura, Lisboa
Marques-Rodrigues_1992	A. H. de Oliveira Marques & Teresa Ferreira Rodrig	Chancelarias Portuguesas : D. João I, vol. III, tomo 3, 1410-1418	1992	3	Instituto Nacional de Investigação Científica / Centro de Estudos Históricos da Universidade Nova de Lisboa, Lisboa
Mascarenhas-Abreu_1879	Joaquim Augusto de Oliveira Mascarenhas & R Clemen	Portugal: diccionario chorographico, historico, heraldico, ethologico, biographico, estatistico, archeologico e bibliographico	1879	1	Pomar, Lisboa
Mascarenhas-Lima_1884	Joaquim Augusto d'Oliveira Mascarenhas & Henrique	Portugal e possessões: novíssimo diccionario chorographico, historico, biographico, archeologico, numismatico, estatistico e heraldino	1883	NA	Manoel Salvador Vieira, Viseu
Mattos_1889	Francisco António de Mattos	Diccionario chorographico de Portugal: parte Continental e Insular	1889	NA	Deposito, Lisboa
Monteiro_1850	José Maria de Souza Monteiro	Diccionario Geographico das Provincias e Possessões Portuguezas no Ultramar; […]	1850	-	Typographia Lisbonense, Lisboa
NA_!!!!	NA	NA	NA	NA	NA
Neves_1961	Carlos Manuel Leitão Baeta Neves	Peixes das águas doces de Portugal	1961	NA	Gazeta das Aldeias, Porto
Neves_1976	Carlos Manuel Leitão Baeta Neves	D. Dinis e um esturjão célebre	1976	NA	Gazeta das Aldeias, Porto
Nobre_1909	Augusto Pereira Nobre	Fauna aquicola de Portugal: peixes, batrachios	1909	-	Imprensa Nacional, Lisboa
NOname_1987	NO NAME	Novo dicionário corográfico de Portugal: actualização	1987	-	-
Pereira_1841a	Agostinho José Pereira	Diccionario geografico do districto administrativo de Lisboa	1841a	1	-
Pereira_1841b	Agostinho José Pereira	Diccionario geografico do districto administrativo de Lisboa	1841b	2	-
Pereira_1861	João Teles Pereira	Compendio de corografia portuguesa	1861	NA	-
Pereira-Rodrigues_1904	Esteves Pereira & Guilherme Rodrigues	Portugal - Diccionario Historico, Chorografico, Heraldico, Biographico, Bibliographico, Numismatico e Artistico (7 Volumes)	1904	1	João Romano Torres - Editor, Lisboa
Pereira-Rodrigues_1906	Esteves Pereira & Guilherme Rodrigues	Portugal - Diccionario Historico, Chorografico, Heraldico, Biographico, Bibliographico, Numismatico e Artistico (7 Volumes)	1906	2	João Romano Torres - Editor, Lisboa
Pereira-Rodrigues_1907	Esteves Pereira & Guilherme Rodrigues	Portugal - Diccionario Historico, Chorografico, Heraldico, Biographico, Bibliographico, Numismatico e Artistico (7 Volumes)	1907	3	João Romano Torres - Editor, Lisboa
Regalla_1888	Francisco Augusto da Fonseca Regalla	Relatorio sobre a Pesca no Rio Minho em 1884	1888	NA	Imprensa Nacional, Lisboa
Rego_1816	José António da Silva Rego	Geographia Moderna de Portugal e Hespanha	1816	NA	Officina de J. F. M. de Campos, Lisboa
Reguart_1791a	António Sañez Reguart	Diccionario Historico de los Artes de la pesca nacional	1791a	1	Imprenta de la Viuda de Don Joaquin Ibarra, Madrid
Reguart_1791b	António Sañez Reguart	Diccionario Historico de los Artes de la pesca nacional	1791b	2	Imprenta de la Viuda de Don Joaquin Ibarra, Madrid
Reguart_1792	António Sañez Reguart	Diccionario Historico de los Artes de la pesca nacional	1792	3	Imprenta de la Viuda de Don Joaquin Ibarra, Madrid
Reguart_1793	António Sañez Reguart	Diccionario Historico de los Artes de la pesca nacional	1793	4	Imprenta de la Viuda de Don Joaquin Ibarra, Madrid
Reguart_1795	António Sañez Reguart	Diccionario Historico de los Artes de la pesca nacional	1795	5	Imprenta de la Viuda de Don Joaquin Ibarra, Madrid
Rodrigues_1844a	António Patrício Pinto Rodrigues	Diccionario Geografico ou Noticia Historica de todas as Cidades, Villas, Rios, Ribeiras, Serras e Portos de Mar dos Reinos de Portugal e Algarve, 10 vols.	1844a	1	Lisboa
Rodrigues_1844b	António Patrício Pinto Rodrigues	Diccionario Geografico ou Noticia Historica de todas as Cidades, Villas, Rios, Ribeiras, Serras e Portos de Mar dos Reinos de Portugal e Algarve, 10 vols.	1844b	2	Lisboa
Rodrigues_1844c	António Patrício Pinto Rodrigues	Diccionario Geografico ou Noticia Historica de todas as Cidades, Villas, Rios, Ribeiras, Serras e Portos de Mar dos Reinos de Portugal e Algarve, 10 vols.	1844c	3	Lisboa
Rodrigues_1844d	António Patrício Pinto Rodrigues	Diccionario Geografico ou Noticia Historica de todas as Cidades, Villas, Rios, Ribeiras, Serras e Portos de Mar dos Reinos de Portugal e Algarve, 10 vols.	1844d	4	Lisboa
Rodrigues_1844e	António Patrício Pinto Rodrigues	Diccionario Geografico ou Noticia Historica de todas as Cidades, Villas, Rios, Ribeiras, Serras e Portos de Mar dos Reinos de Portugal e Algarve, 10 vols.	1844e	5	Lisboa
Rodrigues_1844f	António Patrício Pinto Rodrigues	Diccionario Geografico ou Noticia Historica de todas as Cidades, Villas, Rios, Ribeiras, Serras e Portos de Mar dos Reinos de Portugal e Algarve, 10 vols.	1844f	6	Lisboa
Rodrigues_1844g	António Patrício Pinto Rodrigues	Diccionario Geografico ou Noticia Historica de todas as Cidades, Villas, Rios, Ribeiras, Serras e Portos de Mar dos Reinos de Portugal e Algarve, 10 vols.	1844g	7	Lisboa
Rodrigues_1844h	António Patrício Pinto Rodrigues	Diccionario Geografico ou Noticia Historica de todas as Cidades, Villas, Rios, Ribeiras, Serras e Portos de Mar dos Reinos de Portugal e Algarve, 10 vols.	1844h	8	Lisboa
Rodrigues_1844i	António Patrício Pinto Rodrigues	Diccionario Geografico ou Noticia Historica de todas as Cidades, Villas, Rios, Ribeiras, Serras e Portos de Mar dos Reinos de Portugal e Algarve, 10 vols.	1844i	9	Lisboa
Rodrigues_1844j	António Patrício Pinto Rodrigues	Diccionario Geografico ou Noticia Historica de todas as Cidades, Villas, Rios, Ribeiras, Serras e Portos de Mar dos Reinos de Portugal e Algarve, 10 vols.	1844j	10	Lisboa
Rodriguez-Garcia_1982	Francisco Cantelar Rodríguez & António Garcia y G	Synodicon Hispanum. II, Portugal	1982	2	Biblioteca de autores cristianos, Madrid
Sampaio_1940	M Sampaio	Dicionário corográfico de Portugal	1940	NA	Editoria Portugália, Lisboa
Secco_1853	António Luiz de Sousa Henriques Secco	Memoria historico-chorographica dos diversos concelhos do districto administrativo de Coimbra	1853	NA	Imprensa da Universidade de Coimbra, Coimbra
Serra_1824	José Correia da Serra	Collecção de livros ineditos da historia portuguesa dos reinados de D. Affonso V, a D. João II (5 vol.)	1824	5	Typographia da Academia Real das Sciencias, Lisboa
Silva_1891a	António Artur Baldaque da Silva	Estado Actual das Pescas em Portugal: comprehendendo a pesca maritima, fluvial e lacustre em todo o continente do reino, refreido ao anno de 1886	1891a	1	Imprensa Nacional, Lisboa
Silva_1891b	António Artur Baldaque da Silva	Estado Actual das Pescas em Portugal	1891b	2	Imprensa Nacional, Lisboa
Silveira_1804	Francisco do Nascimento Silveira	Mappa breve da Lusitania antiga, e Galliza bracarense	1804	1	Oficina de Simão Thaddeo Ferreira, Lisboa
Tavares_2009	Maria José Ferro Tavares	As pescas: uma riqueza em extinção?	2009	NA	Caleidoscópio, Lisboa
Teles_1914	Bruno Teles	Sinopse de corografia de Portugal	1914	-	-
Vasconcelos_1884	José Leite de Vasconcelos	Diccionario da chorographia de Portugal contendo a indicação de todas as cidades, villas e freguesias	1884	NA	Livraria Portuense de Clavel & C.ª editores, Porto
Vasconcelos_1926	Augusto de Vasconcelos	Corografia de Portugal	1926	-	-
Vieira_1886a	José Augusto Vieira	Minho Pittoresco	1886a	1	Livraria Antonio Maria Pereira, Lisboa
Vieira_1886b	José Augusto Vieira	Minho Pittoresco	1886b	2	Livraria Antonio Maria Pereira, Lisboa
Visconde_1876	2º Visconde de Vila Maior	O Douro Illustrado: album do rio Douro e paiz vinhateiro	1876	NA	Livraria Universal de Magalhães & Moniz, Porto

**Table 2:** Combination of “Group Name” field and “Sub-group Name” field occurring in the historical records table of the PHish database.

**Group Name**	**Sub-group Name**
*Acipenser sturio*	NA
*Alosa alosa*	NA
*Alosa fallax*	NA
*Alosa* sp.	*Alosa alosa*
*Alosa* sp.	*Alosa fallax*
*Alosa* sp.	NA
*Anaecypris hispanica*	NA
*Anguilla anguilla*	NA
*Carassius auratus*	NA
*Chondrostoma* sp.	*Achondrostoma occidentale*
*Chondrostoma* sp.	*Achondrostoma oligolepis*
*Chondrostoma* sp.	*Iberochondrostoma lemmingii*
*Chondrostoma* sp.	*Iberochondrostoma lusitanicum*
*Chondrostoma* sp.	*Iberochondrostoma sp.*
*Chondrostoma* sp.	NA
*Chondrostoma* sp.	*Pseudochondrostoma duriense*
*Chondrostoma* sp.	*Pseudochondrostoma polylepis*
*Chondrostoma* sp.	*Pseudochondrostoma willkommii*
*Cyprinus carpio*	NA
*Iberochondrostoma almacai*	NA
*Lampetra* sp.	*Lampetra fluviatilis*
*Luciobarbus bocagei*	NA
*Luciobarbus sclateri*	NA
*Luciobarbus* sp.	*Luciobarbus comizo*
*Luciobarbus* sp.	NA
*Mugilidae*	NA
NA	*Alosa* sp.
NA	*Anguilla anguilla*
NA	*Cyprinus carpio*
NA	*Luciobarbus bocagei*
NA	*Luciobarbus* sp.
NA	NA
NA	*Salmo* sp.
*Petromyzontidae*	NA
*Petromyzontidae*	*Petromyzon marinus*
Pleuronectiformes	NA
*Salmo salar*	NA
*Salmo* sp.	*Salmo salar*
*Salmo trutta*	NA
*Salmo trutta*	*Salmo trutta fario*
*Salmo trutta*	*Salmo trutta trutta*
*Salmo trutta fario*	NA
*Salmo trutta trutta*	NA
*Squalius* sp.	*Squalius alburnoides*
*Squalius* sp.	*Squalius aradensis*
*Squalius* sp.	*Squalius carolitertii*
*Squalius* sp.	*Squalius pyrenaicus*
NA–Non-applicable.	

**Table 3:** Fields and respective description contained in each table of the Portuguese Historical Fish Database.

Table	Field	Field description
Basins	WSO_ID	ID of the basin
	Basin Name	Name of the Basin
	B_MidPoint_X	X coordinate of the midpoint of the basin mouth segment
	B_MidPoint_Y	Y coordinate of the midpoint of the basin mouth segment
	Area_B	Area of the Basin
	Perimeter_B	Perimeter of the Basin
Sub-basins	subWSO_ID	ID of the sub-basin
	WSO_ID	ID of the basin
	Sub-basin Name	Name of the sub-basin
	SB_MidPoint X	X coordinate of the midpoint of the sub-basin mouth segment
	SB_MidPoint Y	Y coordinate of the midpoint of the sub-basin mouth segment
	Area_SB	Area of the sub-basin
	Perimeter_SB	Perimeter of the sub-basin
Segments	WSO1_ID	ID of the Segment
	subWSO_ID	ID of the sub-basin
	WSO_ID	ID of the basin
	River	Name of the river
	S_MidPoint_X	X coordinate of the midpoint of the segment
	S_MidPoint_Y	Y coordinate of the midpoint of the segment
	Area_S	Drainage area of the segment
	Length_S	Length of the segment
Taxonomical Groups	Group Name	Name of the taxonomical group
	Sub-group Name	Sub-division or detailed identification within the taxonomical group
	English Name	Common English name
	Portuguese Name	Common Portuguese name
	Other PT Names	Old Portuguese names used
	Phenology	Phenology of the animals in this group
	PT Red List	Portuguese Red List status
	UICN Red List	UICN Red List status
Historical Documents	ID_Doc	ID of the historical document
	Author	Name of the Author of the historical document
	Title	Title of the historical document
	Doc_date	Date or period of the historical document
	Volume	Volume of the historical document
	Publisher_place	Publisher and place of the historical document
	Investigator Name	Name of the investigator that scanned the historical document
	Doc_URL	Hyperlink to the digital document
	Doc_PDF	PDF of the document
	Notes	Notes about the historical document
Historical Records	ID_key	ID of the historical record
	Scale_item	Scale at which to consider the record
	WSO_ID	ID of the basin
	subWSO_ID	ID of the sub-basin
	WSO1_ID	ID of the segment
	Extract Name	Species extract name
	Group Name	Name of the taxonomical group
	Sub-group Name	Sub-division or detailed identification within the taxonomical group
	Village	Historical location of the record
	Basin Name PT	Portuguese name of the basin
	Sub-basin Name	Name of the sub-basin
	River	Name of the river
	Record Date	Date of the historical record
	Century	Century of the historical record
	ID_Doc	ID of the historical document
	Researcher Name	Name of the researcher that scanned this historical document
	Notes	Notes of the historical record
